# Ubiquitin‐Independent Degradation: An Emerging PROTAC Approach?

**DOI:** 10.1002/bies.202400161

**Published:** 2024-11-26

**Authors:** Tiantian Li, Saskia A. Hogenhout, Weijie Huang

**Affiliations:** ^1^ Key Laboratory of Plant Design National Key Laboratory of Plant Molecular Genetics CAS Center for Excellence in Molecular Plant Sciences Chinese Academy of Sciences Shanghai China; ^2^ Department of Crop Genetics John Innes Centre, Norwich Research Park Norwich UK

## Abstract

Targeted protein degradation (TPD) has emerged as a highly promising approach for eliminating disease‐associated proteins in the field of drug discovery. Among the most advanced TPD technologies, PROteolysis TArgeting Chimera (PROTAC), functions by bringing a protein of interest (POI) into proximity with an E3 ubiquitin ligase, leading to ubiquitin (Ub)‐dependent proteasomal degradation. However, the designs of most PROTACs are based on the utilization of a limited number of available E3 ligases, which significantly restricts their potential. Recent studies have shown that phytoplasmas, a group of bacterial plant pathogens, have developed several E3‐ and ubiquitin‐independent proteasomal degradation (UbInPD) mechanisms for breaking down host targets. This suggests an alternative approach for substrate recruitment and TPD. Here, we present existing evidence that supports the feasibility of UbInPD in eukaryotic cells and propose candidate proteins that can serve as docking sites for the development of E3‐independent PROTACs.

## Graphical abstract

The article explores ubiquitin‐independent proteasomal degradation (UbInPD) as an alternative to traditional targeted protein degradation (TPD) using PROTACs. It presents evidence for UbInPD in eukaryotic cells, proposing candidate proteins for developing E3‐independent PROTACs.

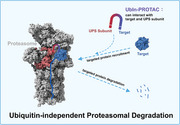

## 1 Introduction

The ubiquitin‐proteasome system (UPS) is the main pathway for targeted protein degradation (TPD) in eukaryotic cells. In this canonical process, proteins earmarked for degradation undergo ubiquitination, a pivotal posttranslational modification that acts as a signal, guiding the targeted proteins to the proteasome for degradation [1]. Protein ubiquitination requires the coordinated actions of E1 activating, E2 conjugating, and E3 ligating enzymes. Specifically, E3 ligases directly interact with their target substrates, providing substrate specificity and determining the specific types of ubiquitin conjugates that are formed. When brought into proximity, E3s were found to catalyze the ubiquitination of unrelated POIs using the cellular ubiquitin machinery. This breakthrough has catalyzed the emergence of the PROTAC technology, representing a revolutionary therapeutic paradigm. By harnessing small molecules for E3‐mediated TPD, PROTAC has illuminated new pathways for therapeutic intervention [2]. Unlike classical enzyme inhibition, which typically functions by blocking the active site of a target protein, PROTAC eliminates the protein entirely from the cellular environment. This provides a more sustained and irreversible approach. Moreover, PROTAC can target proteins considered “undruggable” by traditional small molecules, including scaffolding proteins or those without accessible active sites. By leveraging E3 ligases for substrate degradation, PROTAC also offers differential pharmacology, as it can be tuned for selective degradation, reducing off‐target effects and expanding the range of therapeutic targets [3]. PROTACs hold great potential beyond therapeutics, including biological research for precise protein manipulation, agriculture for enhancing crop traits and protection, and biotechnology for controlling engineered systems. Overall, PROTACs offer a powerful approach to TPD, poised to drive significant advancements across diverse scientific and technological disciplines.

A typical PROTAC molecule consists of three key components: a ligand targeting the POI, a linker, and an E3 ligand. This modular structure enables precise customization and systematic refinement, streamlining the process of drug discovery. Currently, around a dozen PROTAC molecules are progressing through various stages of clinical trials. In addition to PROTAC, other TPD drugs, including immunomodulatory drugs (IMiDs) and selective estrogen receptor degraders (SERDs), have either gained approval or are undergoing clinical evaluation [4]. Despite differences in their mechanisms, all three classes of TPDs induce protein degradation within the proteasome via an E3‐ and ubiquitin‐dependent pathway.

The dependency of TPD drugs on specific E3s poses certain limitations. Even though there are over 600 E3 ligases present in humans, drug design primarily focuses on only a small subset of these, such as Von Hippel‐Lindau (VHL) and Cereblon (CRBN). However, each E3 exhibits distinct expression profiles and enzymatic behaviors toward various protein targets. Consequently, the exclusive targeting of a handful of E3 ligases may not consistently yield optimal efficacy across diverse scenarios [5]. Additionally, protein ubiquitination may be reversed by cellular deubiquitinases, thereby preventing substrate degradation. Another drawback involves the emergence of drug resistance, often associated with mutations or decreased expression of the utilized E3 ligases. To overcome these limitations, there is a growing interest in the functional characterization of additional human E3 ligases that may be suitable for the design of TPD drugs. While there have been some successes, this remains a challenge, primarily due to the need for discovering high‐quality ligands that can effectively bind and recruit these ligases. Beyond discovering new E3 ligases, are there alternative strategies to unlock the complete potential of small‐molecule–driven protein degradation?

**FIGURE 1:**
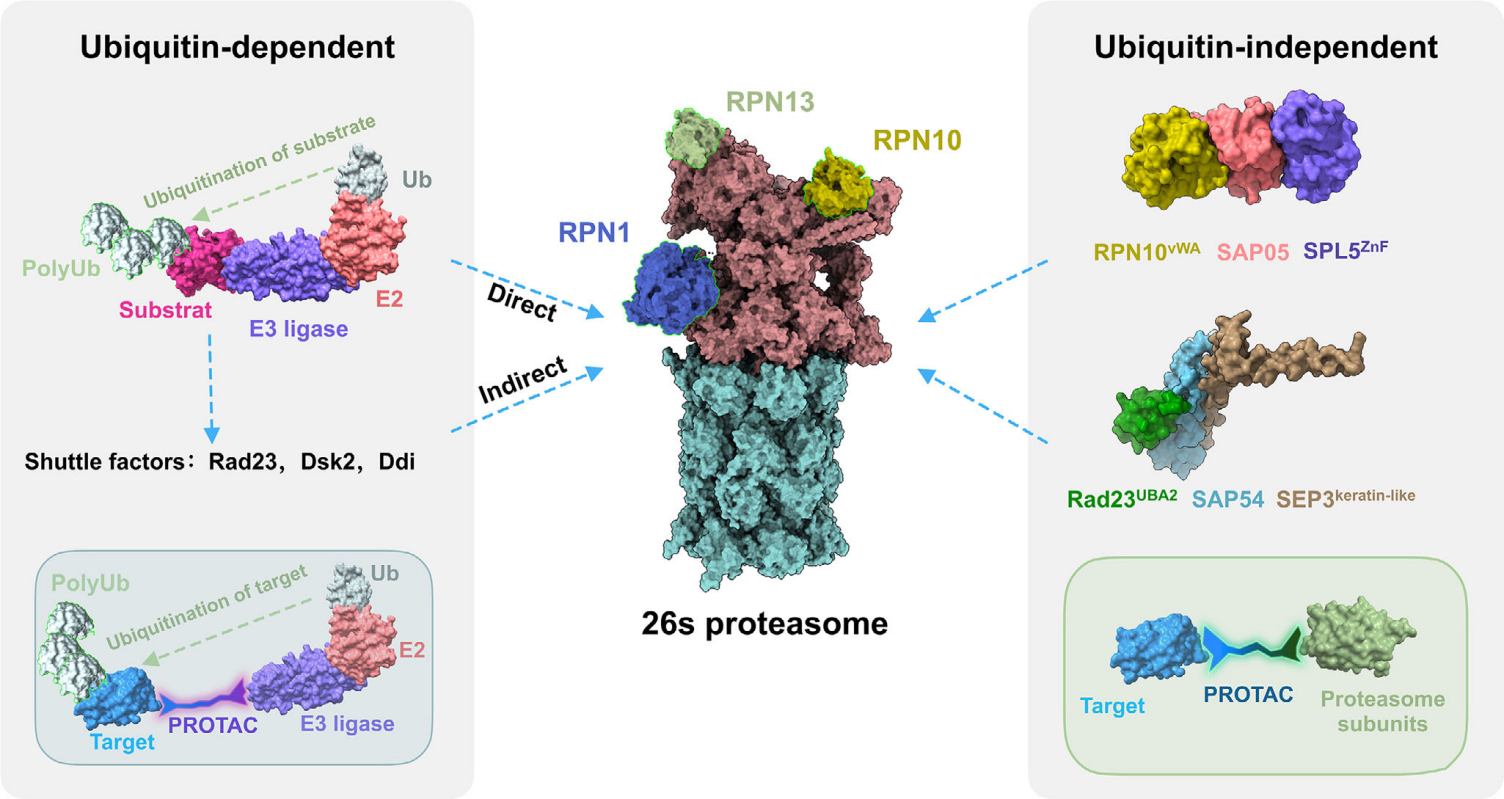
Schematic diagram of ubiquitin‐dependent and ‐independent 26S proteasomal degradation pathways. RPN1, RPN10, and RPN13 are ubiquitin receptors on the 26S proteasome, whereas Rad23, Dsk2, and Ddi are shuttle factors that deliver ubiquitinated cargo to the proteasome via engaging with the ubiquitin receptors. Phytoplasma effectors SAP05 and SAP54 were discovered to directly bind distinct domains of 26S proteasome proteins. SAP05 binds to the RPN10 vWA domain, while SAP54 interacts with the Rad23 UBA domains. This interaction facilitates the ubiquitin‐independent degradation of SPL transcription factors by targeting their ZnF domains, exemplified by SPL5^ZnF^, and the keratin‐like domain of MADS‐box transcription factors, exemplified by SEP3^keratin‐like^. The RPN10^vWA^‐SAP05‐SPL5^ZnF^ structure was modeled based on crystal structures of binary complexes [9]. The Rad23^UBA2^‐SAP54‐SEP3^keratin‐like^ complex was modeled using AlphaFold3 [14]. The Ub‐dependent PROTAC and Ub‐independent PROTAC approaches are illustrated in the insets. UBA indicates ubiquitin‐associated; ZnF, zinc‐finger.

Exploring the intricate mechanisms utilized by bacterial plant pathogens, especially those thriving within the cytoplasm of their eukaryotic host cells like phytoplasmas, can unveil valuable insights. Phytoplasmas are renowned for their ability to induce a spectrum of extravagant developmental alterations in their host plants. Diseased plants often exhibit rampant growth, lose their ability to reproduce, and become prime reservoirs for spreading diseases [6, 7]. Phytoplasma employ a sophisticated strategy, releasing virulence proteins, also known as effectors, into plants to manipulate their development. Fascinatingly, multiple phytoplasma effectors converged on hijacking the 26S proteasome, a conserved protease complex in eukaryotes. This complex orchestrates the degradation of host targets through a ubiquitin‐independent pathway (Figure 1). The phytoplasma effector SAP05, for instance, interacts with a spectrum of plant SPL and GATA transcription factors, alongside the key plant 26S proteasome subunit RPN10—a central ubiquitin receptor. This interaction culminates in the assembly of ternary complexes, facilitating direct substrate recruitment without the necessity for ubiquitination [8, 9, 10, 11]. Another effector, SAP54, exploits the shuttle protein RADIATION SENSITIVE23 (Rad23) to recruit nonubiquitinated floral MADS‐box transcription factors for proteasomal destabilization [12, 13]. The discovery of these noncanonical proteolytic pathways employed by microbial pathogens offers an opportunity to utilize an alternative avenue for the development of TDP molecules, bypassing the conventional reliance on E3 ligases and ubiquitin.

## 2 Evidence for Ubiquitin‐Independent Proteasomal Degradation (UbInPD)

**FIGURE 2:**
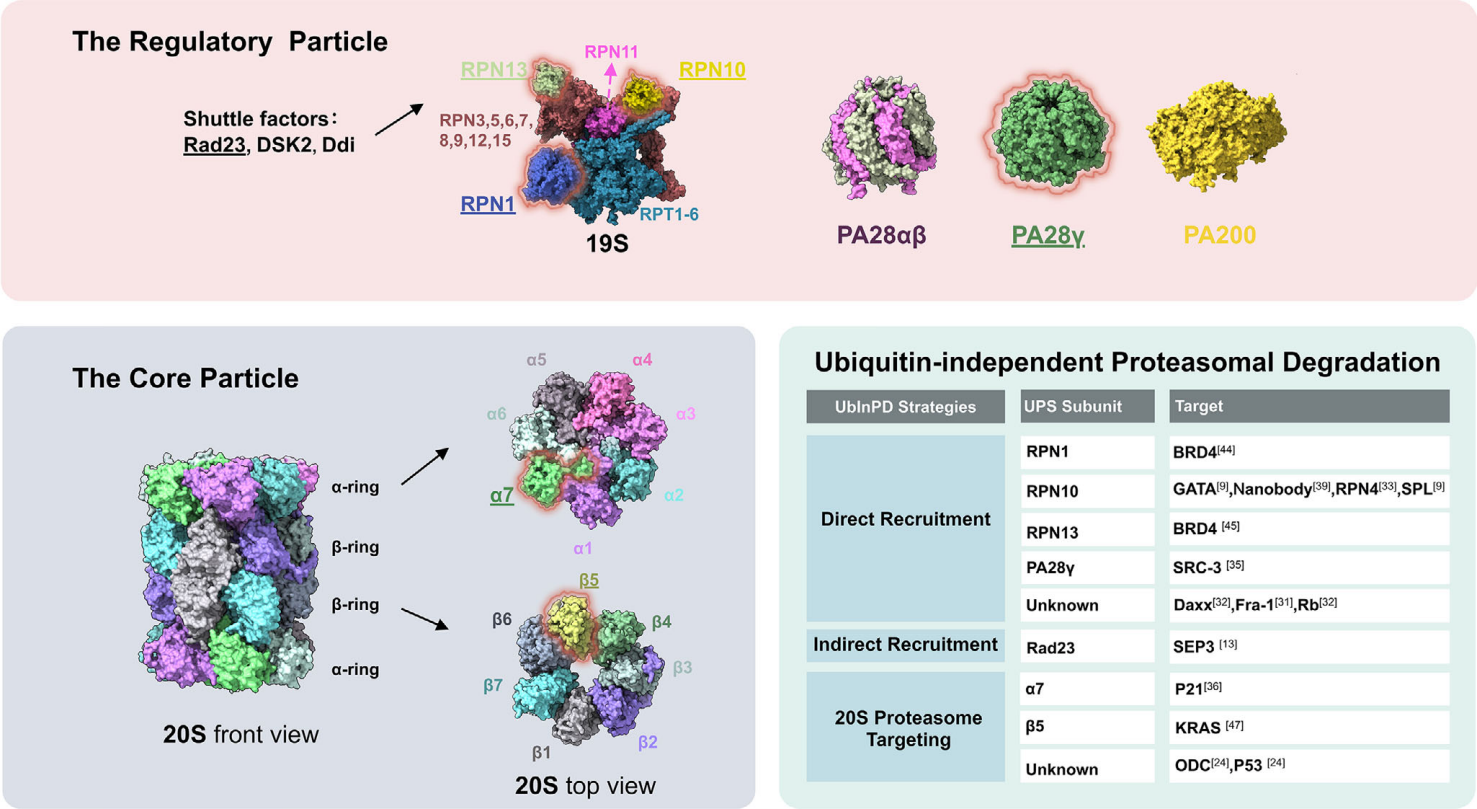
Components of the 26S proteasome involved in UbInPD. In different eukaryotic cells, the 20S proteasome can be uncapped or capped by various regulatory complexes. These regulatory complexes include the 19S regulatory particle, PA200, the heptameric hetero PA28αβ ring, or the homo PA28γ ring. The proteasome subunits known to be involved in facilitating UbInPD are highlighted. The UbInPD strategies are classified into three main groups based on different recruitment methods, as shown in the lower right panel. UbInPD indicates ubiquitin‐independent proteasomal degradation.

The targeting of a protein for proteasomal degradation requires a two‐component degron on the substrate, consisting of a proteasome binding signal and a degradation initiation region [15, 16]. In addition to acting as a proteasomal localization signal, site‐specific ubiquitination, and ubiquitin unfolding can trigger conformational changes in the proteasome, essential for its activation. For certain well‐folded proteins, ubiquitination may also generate the unstructured regions required to initiate degradation [17, 18, 19]. Depending on the organisms and cell types, the core particle of the proteasome (CP; 20S) can be uncapped, capped at one or both ends by the 19S regulatory particle (RP; 19S), or other regulatory particles to form distinct populations that are separately stable in cells (Figure 2). Both the uncapped CP and the capped CP can carry out protein degradation [20, 21]. Structurally, the cylindrical CP is formed by four stacked rings (α7β7β7α7). The outer α rings function as a gateway, allowing substrates to enter, while the inner β rings house the proteolytic sites. The 19S RP can be separated into two components: the base and the lid. The base contains a ring of AAA‐ATPases (RPT1‐6) that directly interact with the CP, as well as four non‐ATPase subunits (RPN1, 2, 10, and 13). The lid is composed of nine non‐ATPase subunits (RPN3, 5, 6, 7, 8, 9, 11, 12, and 15). The RP sits on top of the CP's α ring, capturing and preparing substrates for degradation [22]. Typically, covalently attached polyubiquitin chains serve as the proteasomal binding signal, which can be recognized by proteasomal ubiquitin receptors, including RPN1, RPN10, and RPN13 on the 19S RP, either directly or indirectly with the help of proteasome shuttle factors, such as Rad23, Dsk2, and Ddi1 [23]. However, there are also multiple evidence for the existence of ubiquitin‐independent proteasomal recruitment, leading to ubiquitin‐independent degradation.

UbInPD was first observed within the 20S proteasome, where proteins with intrinsically disordered regions are degraded without the need for ubiquitin tagging. Some examples of such proteins include the enzyme ornithine decarboxylase (ODC), the cell cycle regulators p53 and p21 [24, 25]. In the case of p21, it contains a degradation signal in its C‐terminus, and its association with the proteasome α7 subunit is facilitated by the accessory protein MDM2, leading to its ubiquitin‐independent degradation by the 20S proteasome [26, 27]. More broadly, the 20S proteasome‐mediated UbInPD pathway is linked to the elimination of oxidized proteins under various stress conditions [28]. Intrinsically disordered proteins, misfolded proteins, or those that expose unstructured regions due to oxidation, mutation, or protein–protein interactions, are often subject to proteolysis in the 20S proteasome in a ubiquitin‐independent manner [29]. The unstructured regions of these substrate proteins play a crucial role in facilitating their binding and translocation within the 20S proteasome. Accordingly, many of the substrate proteins are known to directly bind to specific subunits of the 20S proteasome.

UbInPD within the 26S proteasome had received less attention until more recently. Earlier studies have documented the involvement of several 19S proteasomal subunits in the ubiquitin‐independent degradation of various proteins, including the oncoprotein Fra‐1, the tumor suppressor retinoblastoma (Rb), death domain associated protein (Daxx), ODC, and the proteasome biogenesis regulator RPN4 [30, 31, 32, 33]. A recent systematic study revealed that UbInPD is more prevalent than currently appreciated, and the in vivo degradation of UbInPD substrates mainly depends on 26S proteasomes and the shuttle factor Dsk2, also known as ubiquilins [34]. In addition to the 19S RP, other proteasome activators, such as PA28γ, can also mediate the UbInPD of many substrates [35, 36]. Moreover, a recently discovered mammalian protein called midnolin has been shown to associate with the proteasome and promote the degradation of many short‐lived transcription factors through a mechanism that is independent of ubiquitination. However, the exact way in which midnolin recruits the substrates to the proteasome is not yet clear [37]. Notably, both ubiquitin‐dependent degradation and different forms of UbInPD often act on the same protein to regulate its abundance. Many of these proteins that undergo UbInPD play significant roles in cellular growth control, cell cycle progression, and/or oncogenesis, which underscores the importance of the UbInPD pathway.

## 3 Direct Proteasomal Recruitment as a Viable Strategy for TPD

As previously noted, current TPD strategies predominantly rely on ubiquitin‐dependent proteasomal degradation. This raises the question of whether alternative UbInPD mechanisms could be explored as additional solutions. UbInPD mechanisms have been witnessed during the infection cycle of various oncogenic viruses. Here, viral proteins strategically target host proteins implicated in oncogenesis and tumor suppression, instigating their degradation [38]. The discovery that phytoplasma effectors commandeer either the proteasomal receptor RPN10 or the shuttle factor Rad23 for TPD highlights a significant evolutionary adaptation in pathogens. It suggests that pathogens have independently evolved to exploit ubiquitin‐independent TPD strategies. There is compelling evidence substantiating the utilization of RPN10 and Rad23 as docking sites for recruiting non‐ubiquitinated proteins. For instance, a nanobody that recognizes a short epitope on the human RPN10 protein is consistently broken down by the 26S proteasome in a way that does not depend on ubiquitination [39]. Noteworthy, the epitope that the nanobody recognizes is located within the C‐terminal region of RPN10, while SAP05 binds specifically to its N‐terminal vWA domain, indicating that different surface areas on RPN10 can serve as the docking sites. Meanwhile, inducible systems that relies on rapamycin‐driven protein–protein interaction involving the target protein and the ubiquitin‐like (UbL) domain of Rad23 or RPN10 has also been demonstrated to induce UbInPD in mammalian cells and yeasts, respectively [40, 41]. Interestingly, both RPN10 and Rad23 avoid being degraded by the proteasome because they lack specific regions that initiates the proteasomal degradation process. However, the addition of non‐specific, unstructured tails to these proteins promotes their degradation [42, 43]. Similarly, for native proteins that typically do not display degradation initiation regions, the binding of chimeric molecules may adjust their positioning relative to proteasome subunits, aiding in their translocation. Therefore, nonubiquitinated cellular proteins are amenable for UbInPD if these substrates can be recruited to the proteasome by binding to certain proteasome subunits, such as RPN10 and Rad23.

Emerging proof‐of‐concept studies have shown the feasibility of TPD via direct proteasome recruitment. Bashore and colleagues identified a potent peptidic macrocycle that binds directly to one of the ubiquitin receptors RPN1 (PSMD2). When this macrocycle is conjugated to a BRD4 ligand, the resulting heterobifunctional molecule enables the degradation of BRD4 in cells without the requirement for its ubiquitination [44]. In this case, the macrocycle epitope is positioned adjacent to the AAA‐ATPase ring of the proteasome, facilitating the delivery of the target protein. Likewise, two groups use the other ubiquitin receptor RPN13 as the docking site. In one study, the authors designed bifunctional molecules that connect an RPN13‐binding ligand and a BRD4‐binding ligand using PEG linkers of varying lengths. These bifunctional molecules enabled the UbInPD of BRD4 in vivo [45]. In another study, the researchers fused a HaloTag to the RPN13 proteasome subunit. When cells were exposed to a chimeric molecule consisting of a HaloTag ligand and a bromodomain ligand, they observed degradation of the BRD2 protein without the need for ubiquitination [46]. However, BRD4, which also binds to the chimeric molecule and can be recruited to the proteasome, was not degraded, suggesting that additional requirements are necessary for effective degradation. Another chemical knockdown approach, Chemical knockdown with Affinity aNd Degradation DYnamics (CANDDY), was developed to facilitate the degradation of undruggable proteins via linking modified proteasome inhibitors to substrate‐binding molecules. For instance, the proteasome inhibitors MLN2238/9708 are modified and attached to a Ras inhibitor, creating a molecule that mediates KRAS degradation in a ubiquitin‐independent manner [47]. Furthermore, despite the absence of the ubiquitin system in bacteria, selective protein degradation using a PROTAC approach has been shown. The small‐molecule degraders, so‐called BacPROTACs, bring targets to the substrate receptor of the ClpC:ClpP protease thereby enabling degradation [48]. The similarity between UbInPD and BacPROTACs further supports the notion that TPD via direct recruitment can be adapted across different protein degradation systems.

In summary, UbInPD strategies can be categorized into three primary types based on their mechanisms (Figure 2). The first category, direct recruitment, involves the direct interaction of the target protein with proteasomal subunits, such as ubiquitin receptors (RPN1, RPN10, RPN13) or the PA28γ activator. The second category, indirect recruitment, depends on intermediary factors or shuttle proteins, like Rad23 and Dsk2, which facilitate the interaction between the target protein and the proteasome. The third category, 20S proteasome targeting, involves engaging the 20S proteasome's catalytic core directly, thereby bypassing the 26S proteasome complex altogether. As research continues to advance, it is likely that additional components within the UPS‐26S proteasome system or other proteins yet to be identified may also mediate UbInPD. The successful application of this strategy in various proof‐of‐concept studies highlights its versatility and effectiveness, underscoring the need for further exploration. For subunits that lack known small‐molecule ligands (such as RPN10 or Rad23), ligand discovery efforts would be required. This could involve high‐throughput screening or structure‐based drug design based on the available structural data of these subunits. Meanwhile, chemical proteomics approaches can be used to identify additional proteasomal subunits or unknown proteins involved in UbInPD.

## 4 Conclusions

UbInPD represents a promising frontier in TPD, expanding the toolkit for targeting and regulating proteins across a wide range of scientific and therapeutic contexts. Incorporating UbInPD into PROTAC strategies could thus enhance their versatility, effectiveness, and scope, potentially leading to more robust and targeted therapeutic and research applications. We anticipate that more proteasome subunits can be leveraged as docking sites for small molecules, enabling the direct recruitment of target proteins for degradation (Figure 2). However, most chimeric degradation molecules developed so far demonstrate only moderate efficacy in inducing target protein degradation. Further efforts will be necessary to evaluate and improve the effectiveness of such degrader molecules. Gaining a deeper understanding of substrate relocation mechanisms is crucial for the rational design of small molecules with optimal binding properties and desired characteristics. In this context, detailed analyses of the mechanisms by which pathogen proteins degrade targeted proteins via UbInPD pathways will provide valuable insights. Additionally, advances in artificial intelligence and machine learning can greatly assist in the design of UbInPD molecules. By integrating these AI‐powered approaches with a better understanding of the underlying biology, researchers can accelerate the development of highly effective and selective UbInPD‐based PROTACs for therapeutic applications.

## Author Contributions

All authors contributed to the writing of this manuscript.

## Conflict of Interest

The authors declare no conflicts of interest.

## Data Availability

Data sharing not applicable as no new data generated.
